# Comparative transcriptome analysis reveals genes associated with variation in liver copper concentration in Polish Merino sheep

**DOI:** 10.1038/s41598-025-04330-8

**Published:** 2025-06-04

**Authors:** Olusegun Olaniyi Adeniyi, Ewa Grochowska, Zexi Cai, Gesine Lühken

**Affiliations:** 1 Institute of Animal Breeding and Genetics, Justus Liebig University, 35390 Giessen, Germany; 2 Department of Oncology, Faculty of Health Sciences, Collegium Medicum, Nicolaus Copernicus University, Łukasiewicza 1 St., 85-821 Bydgoszcz, Poland; 3 Center for Quantitative Genetics and Genomics, Aarhus University, C. F. Møllers Allé 3, Aarhus, Denmark

**Keywords:** Transcriptome analysis, Genes, Liver, Copper concentration, Sheep, Transcriptomics, Gene expression

## Abstract

Economic gain for sheep farmers is continually affected by incidences of copper intoxication and deficiency. This study assessed the ovine genome for differentially expressed genes (DEGs) influencing within-breed variation in hepatic copper content in Polish Merino sheep. To this end, we compared whole genome RNA sequence expression data from 12 female Polish Merino sheep liver samples (dataset 1) selected and grouped equally into high liver copper (HLC) and low liver copper (LLC) groups. Likewise, dataset 2 (a subset of dataset 1) containing 6 samples (HLC = 3; LLC = 3) was analyzed. The DEG analysis of HLC vs LLC groups for both data sets revealed a lower number of significant DEGs for dataset 1 (n = 64) when compared to dataset 2 (n = 252), respectively. Of the significant DEGs, 53 were jointly identified with both datasets. Identified DEGs downregulated in the HLC group including *MVD*, *DHCR7*, *LOC101113583* and *ERG28* are implicated in cholesterol synthesis, whereas *AKR1B10* and *CYP26A1* are associated with retinoic acid synthesis. The cholesterol biosynthetic process (GO:0006695) and the retinol metabolism (oas00830) were also observed by analysis of enriched gene ontologies, and Kyoto encyclopedia of genes and genomes pathways, respectively. Our findings provide new information on candidate genes associated with within-breed differences in hepatic copper concentration.

**Supplementary Information:**

The online version contains supplementary material available at 10.1038/s41598-025-04330-8.

## Introduction

Sheep are more susceptible to copper (Cu) intoxication when compared with other ruminants such as cattle and goats^1,2^. Cu is an important trace mineral that is involved in many biological processes as a part of many copper-dependent enzymes including cytochrome c oxidase (CCO), ferroxidase II, tyrosinase, superoxide dismutase 1 (SOD1) and superoxide dismutase 2 (SOD2) that play essential roles in mitochondrial respiration, iron oxidation, pigmentation and anti-oxidation of free radicals, respectively^3^.

Within-breed variability of hepatic Cu levels, under the same feeding condition, have been reported in earlier studies^4–6^. Sivertsen and Løvberg^6^ found Cu concentration ranges of 89–221 µg/g wet weight (ww) and 87–186 µg/g ww in the livers of Norwegian Dala ewes kept on pasture at different seasons. Recently, we published a work that supported the within-breed variability in liver Cu content in Merinoland sheep^7^. In this report, hepatic Cu ranged from 67–273 mg/kg dry matter (DM) and 20–188 mg/kg DM in Merinoland sheep from two herds kept in Bavaria. In addition to the variability in liver Cu concentration, we estimated a heritability of 0.67 (s.e 0.29) for this trait^7^. This is comparable to the heritability of 0.60 (s.e 0.32) estimated by Judson et al.^8^ in Merino sheep. All these findings point to a significant influence of genetic factors.

Existing documentation provides evidence of the involvement of some genes in hepatic Cu transport and homeostasis^2,9^. Among them are cyclooxygenase 17 (*COX17*), copper transporter 1 (*CTR1*), copper chaperone for superoxide dismutase (*CCS*), antioxidant-1 (*ATOX1*), metallothionein (*MT*), copper-transporting ATPase 2 (*ATP7B*), cytochrome c oxidase (*CCO*) and superoxide dismutase 1 (*SOD1*). The *CTR1*, *ATOX1* and *ATP7B* genes function in import, transport and efflux of hepatic Cu into, within and out of hepatocytes, respectively^10^*.* Other Cu-related genes including *CCS* and *COX17* are involved in Cu transport to SOD1 and CCO target proteins, respectively^9^.

In recent years, advancement in high-throughput technologies such as DNA microarray and RNA sequencing (RNA-seq) have been keys in the development of methods used to measure genome-wide gene expression patterns^11^. Previous studies on the expression of genes associated with hepatic Cu levels have revealed potential candidate genes associated with differences in liver Cu concentration^12–14^. Using human HepG2 cells and microarray analysis, Muller et al.^12^ reported changes in gene expression patterns of liver cells after Cu overload. Furthermore, the authors found that Cu overload in liver cells resulted in the upregulation of genes such as *MT1A, HMOX1, TXNRD1, IL8* and *GCLM* involved in heavy-metal detoxification, oxidative stress, electron transport, signaling and glutathione biosynthesis, respectively^12^. In addition to genome-wide transcriptome analysis, the application of RNA-seq permits the use of small sample sizes with the minimum recommended value ranging from 3 to 6 replicates per condition^15,16^. Previous transcriptome analysis studies using ovine hepatic tissue have been reported with the identification of differentially expressed genes (DEGs) associated with odour and flavour^17^, fatty acid metabolism^18^, and lamb tenderness^19^. Similarly, a recent study by Jin et al.^20^ using RNA-seq technology revealed that genes including *ACTN2* and *GHRHR* were upregulated during Cu supplemented feeding, while *FOXO3* and *TPSB2* were upregulated during Cu deficient feeding of Wu Ranke sheep. In that study, expression patterns of liver cells from sheep fed deficient or supplemented Cu were compared with sheep on a control diet.

In our study, we hypothesized that gene expression in hepatic tissue with low and high Cu content from sheep kept under similar feeding and environmental conditions differ significantly. For testing this hypothesis, we analyzed liver Cu content of Polish Merino sheep kept under similar treatment conditions. Liver samples of selected sheep with the lowest and highest liver Cu concentration were compared in a genome-wide gene expression study to identify differences in gene expression patterns associated with varying liver Cu levels in Polish Merino sheep.

## Results

### Statistics of liver Cu concentration and age

After analysis of samples for hepatic Cu content, our results show that liver Cu concentration for all samples ranged from 62.00 to 522.61 mg/kg DM with a mean hepatic Cu concentration of 231.07 mg/kg DM. Mean liver Cu concentration of samples in the low liver Cu (LLC) group and samples in the high liver Cu (HLC) group for dataset 1 were 130.12 and 372.76 mg/kg DM, respectively (Table 1). For dataset 2, average liver Cu levels for the LLC and HLC groups were 100.19 and 318.45 mg/kg DM, respectively. Moreover, a significant difference (*P* < 0.05) in means between the LLC and HLC groups was observed for dataset 1 (Table 1). Regarding the age, we observed a range of 250 to 315 days with a mean of 288 days (Table 1) for all samples. Table 1 shows that the ages of sampled sheep in the LLC groups for datasets 1 and 2 averaged 282.83 and 296.33 days, respectively. On the other hand, mean values for animal ages in the HLC groups for datasets 1 and 2 were 304.17 and 299.67 days, respectively. Analysis for dataset 1 shows that ages of the LLC group were significantly lower than that of the HLC group (*P* < 0.05) (Table 1).

**Table 1:** Descriptive and inferential statistics of liver Cu concentration and animal age of complete dataset and liver copper (LC) groups for datasets 1 and 2. LLC = low liver copper; HLC = high liver copper.

Sample groups	Number	Liver Cu concentration				Age			
		Mean Cu (mg/kg DM) ± SEM	Min	Max	*P*-value	Mean (age in days)	Min	Max	*P*-value
All	38	231.07 ± 13.70	62.00	522.61		288.00 ± 3.29	250.00	315.00	
Dataset 1									
LLC	6	130.12 ± 16.17	62.00	179.69	0.00	282.83 ± 7.38	264.00	309.00	0.04
HLC	6	372.76 ± 35.60	296.39	522.61		304.17 ± 4.83	278.00	311.00	
Dataset 2									
LLC	3	100.19 ± 13.48	62.00	142.58		296.33 ± 6.47	274.00	309.00	
HLC	3	318.45 ± 6.40	296.39	331.46		299.67 ± 6.26	278.00	311.00	

### Statistics of the RNA sequencing and mapping summary

A total of 12 samples (LLC = 6 and HLC = 6, respectively) were sequenced using the Illumina NovaSeq 6000 sequencing platform. After filtering for adapter sequences and low-quality reads, the mean number of clean reads generated from LLC and HLC groups were 53898532 and 54607901, respectively (Supplementary Table S3). In addition, we observed a minimum of 98.32% for Q20 bases and 93.86% for Q30 bases with the percentage GC content ranging from 44.18 to 46.16 for all samples. The percentage of the clean reads uniquely mapped to the ovine reference genome (NCBI: *Ovis aries*, ARS-UI_Ramb_v2.0, annotation release 105) ranged from 91.46% to 95.2% for the LLC and 91.87% to 95.95% HLC groups (Supplementary Table S3), respectively.

### Principal components

**Fig. 1:**
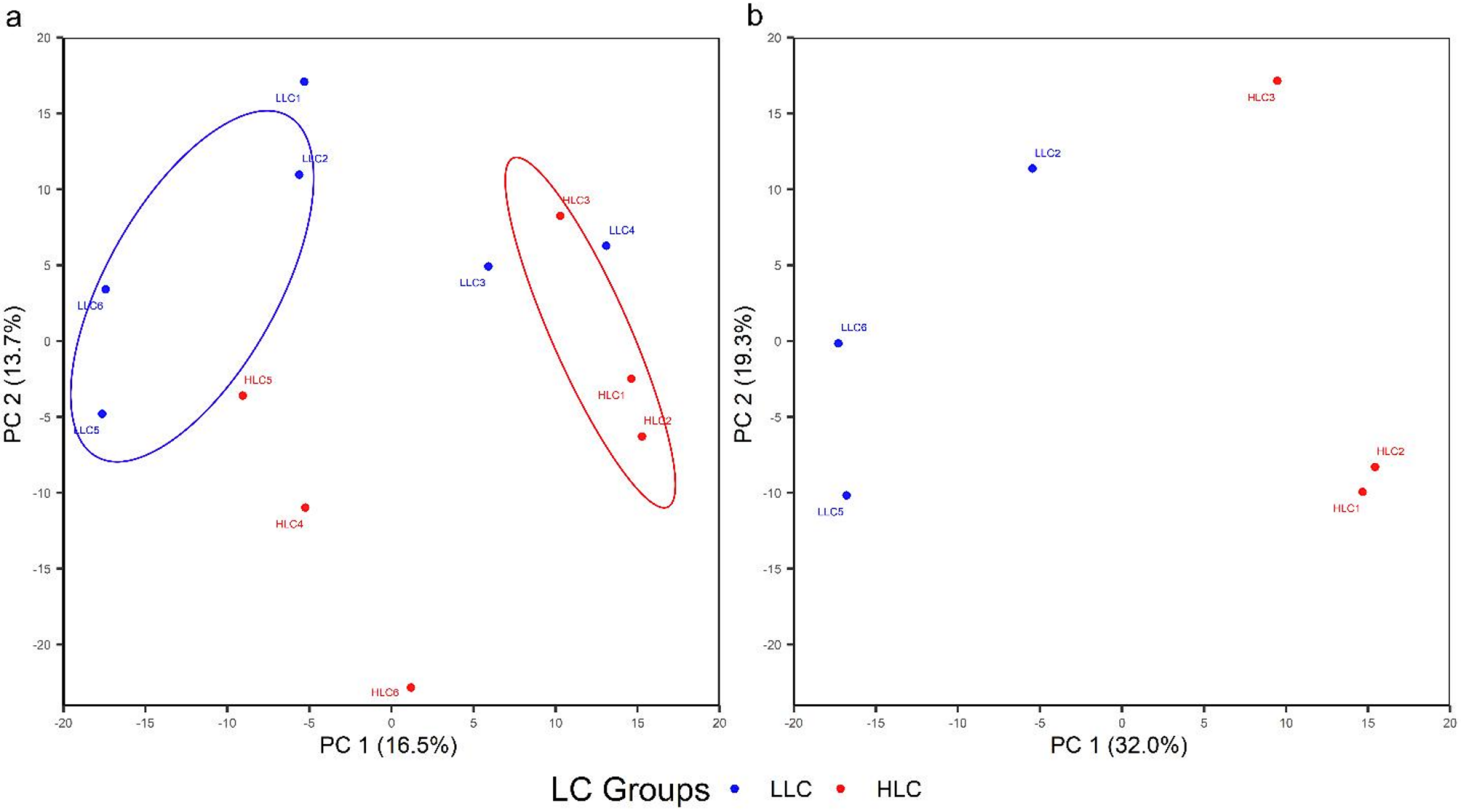
PCA plots of principal component 1 (PC 1) vs PC 2 for datasets 1 (**a**) and 2 (**b**). LC = liver copper; LLC = low liver copper; HLC = high liver copper; encircled samples = samples selected into dataset 2.

The principal component analysis derived from the RNAseq reads counts was used to examine the cluster of samples. The principal component 1 (PC1) and PC2 for dataset 1 explained approximately 16.5% and 13.7% of the variability between samples, respectively (Fig. 1a). Although the PC1 did not separate the samples into distinct groups (LLC and HLC) in the PCA plots, the PC2 separated most of the samples according to their liver Cu (LC) groups. Using dataset 2, a second PCA plot showing PC1 and PC2 was constructed with samples clearly separated by PC1 which accounted for 32% of the sample variation (Fig. 1b). Moreover, PC2 explained 19.3% of the sample difference for dataset 2. Of the two PCA plots, the PCA plot for dataset 2 showed the better clustering of samples into LC groups (Figs. 1a and 1b).

### Identified DEGs, enriched functions and protein–protein interaction network.

**Fig. 2:**
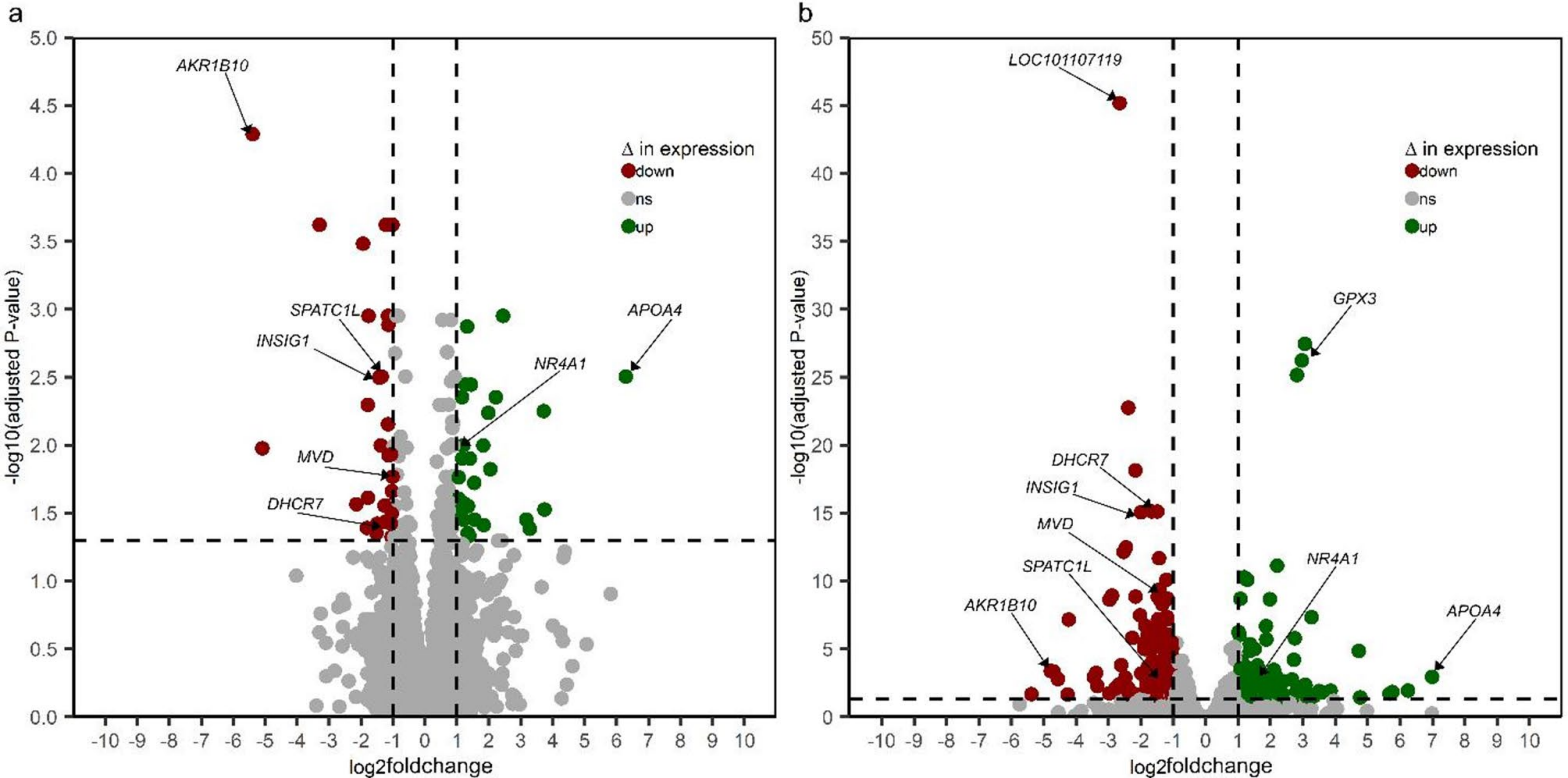
Volcano plots of significantly (FDR ≤ 0.05; |log2foldchange|≥ 1) up- or down-regulated differentially expressed genes (DEGs) in the high liver copper (HLC) group for datasets 1 (**a**) and 2 (**b**).

**Fig. 3:**
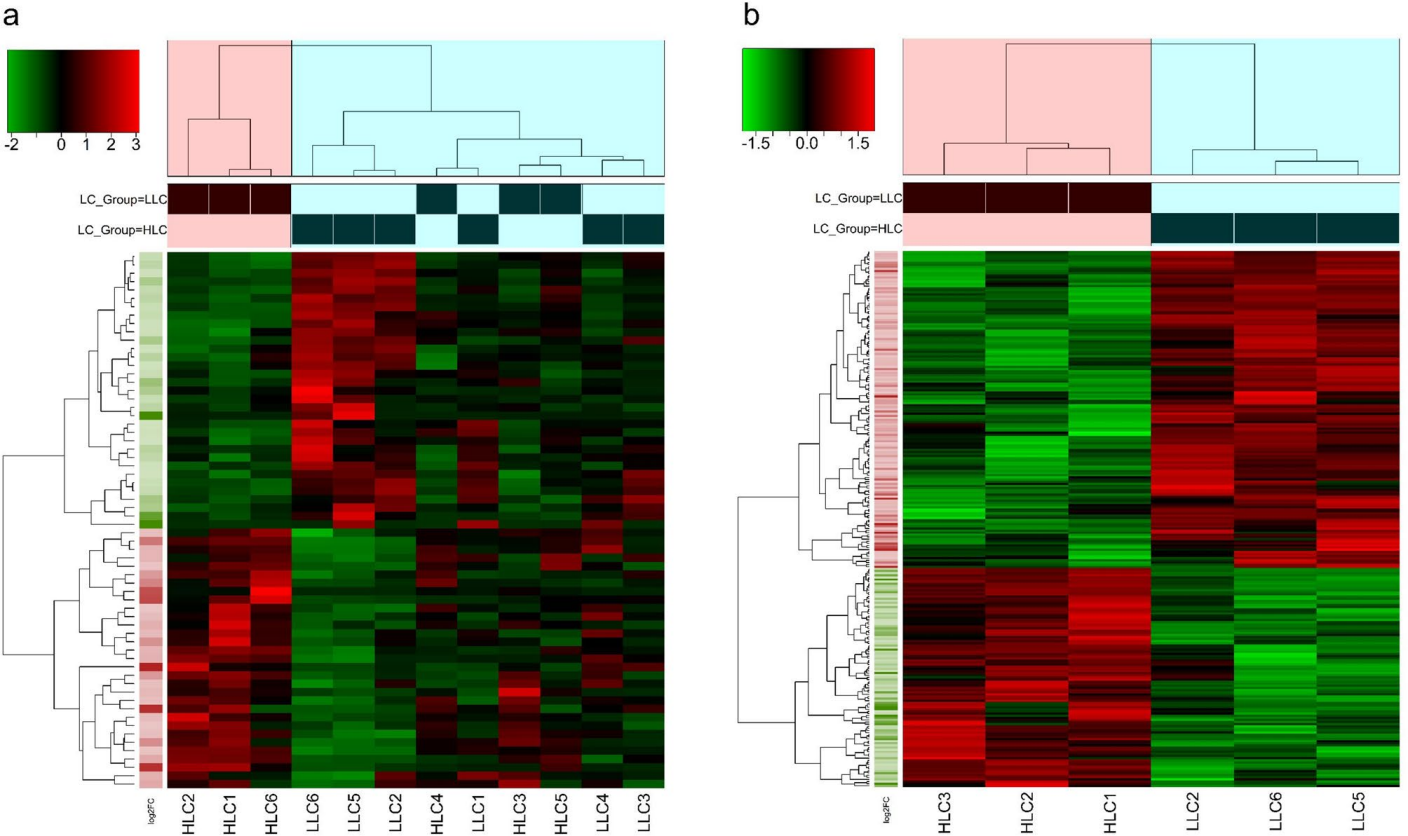
Heatmap plots of the degree of expression of significantly (FDR ≤ 0.05; |log2foldchange (log2FC)|≥ 1) up- or down-regulated differentially expressed genes (DEGs) for datasets 1 (**a**) and 2 (**b**). LC = liver copper; LLC = low liver copper; HLC = high liver copper.

**Fig. 4:**
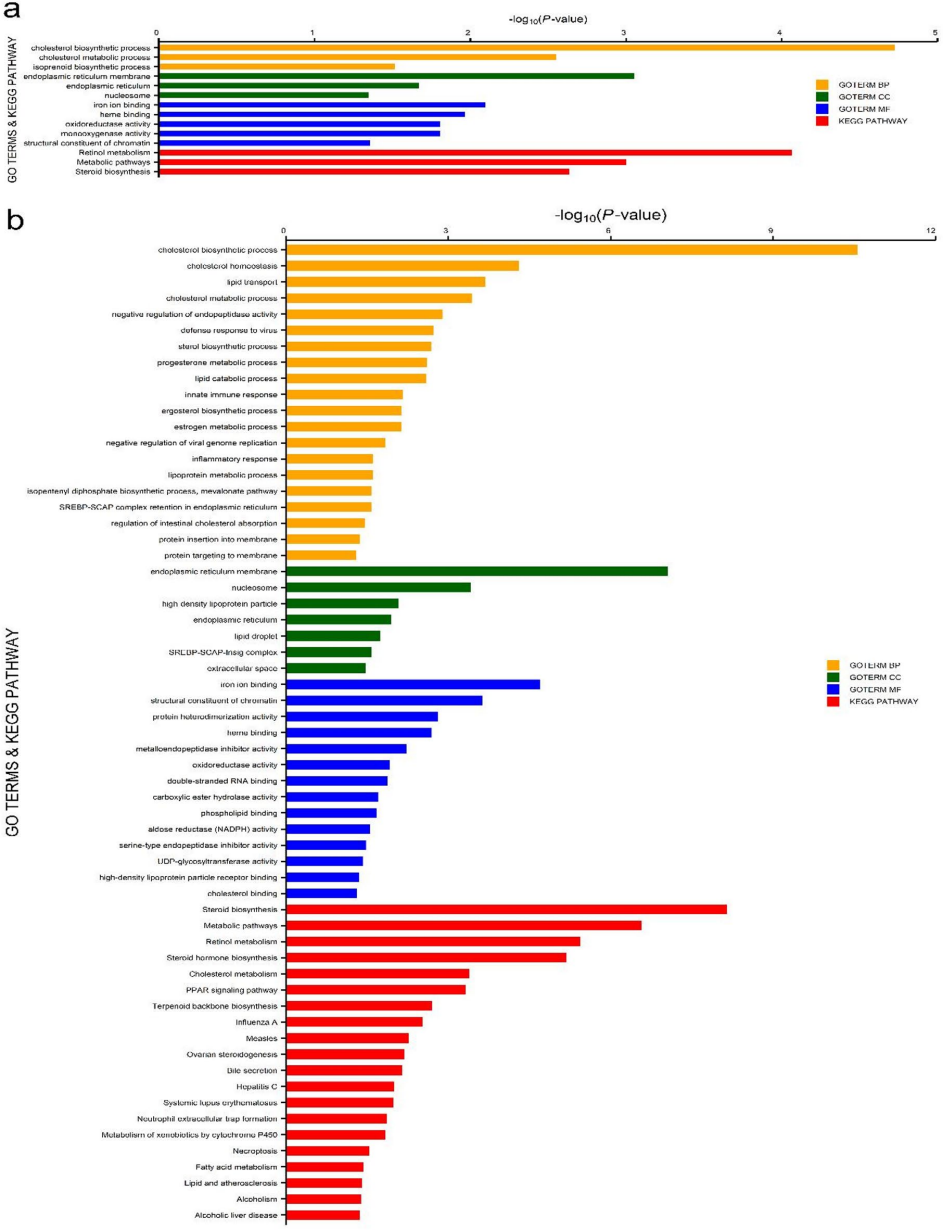
Enriched Gene Ontology (GO) and Kyoto encyclopaedia of genes and genomes (KEGG) pathway terms of significantly up- or down-regulated DEGs for datasets 1 (**a**) and 2 (**b**). GOTERMs: BP = biological process; CC = cellular component; MF = molecular function.

**Fig. 5:**
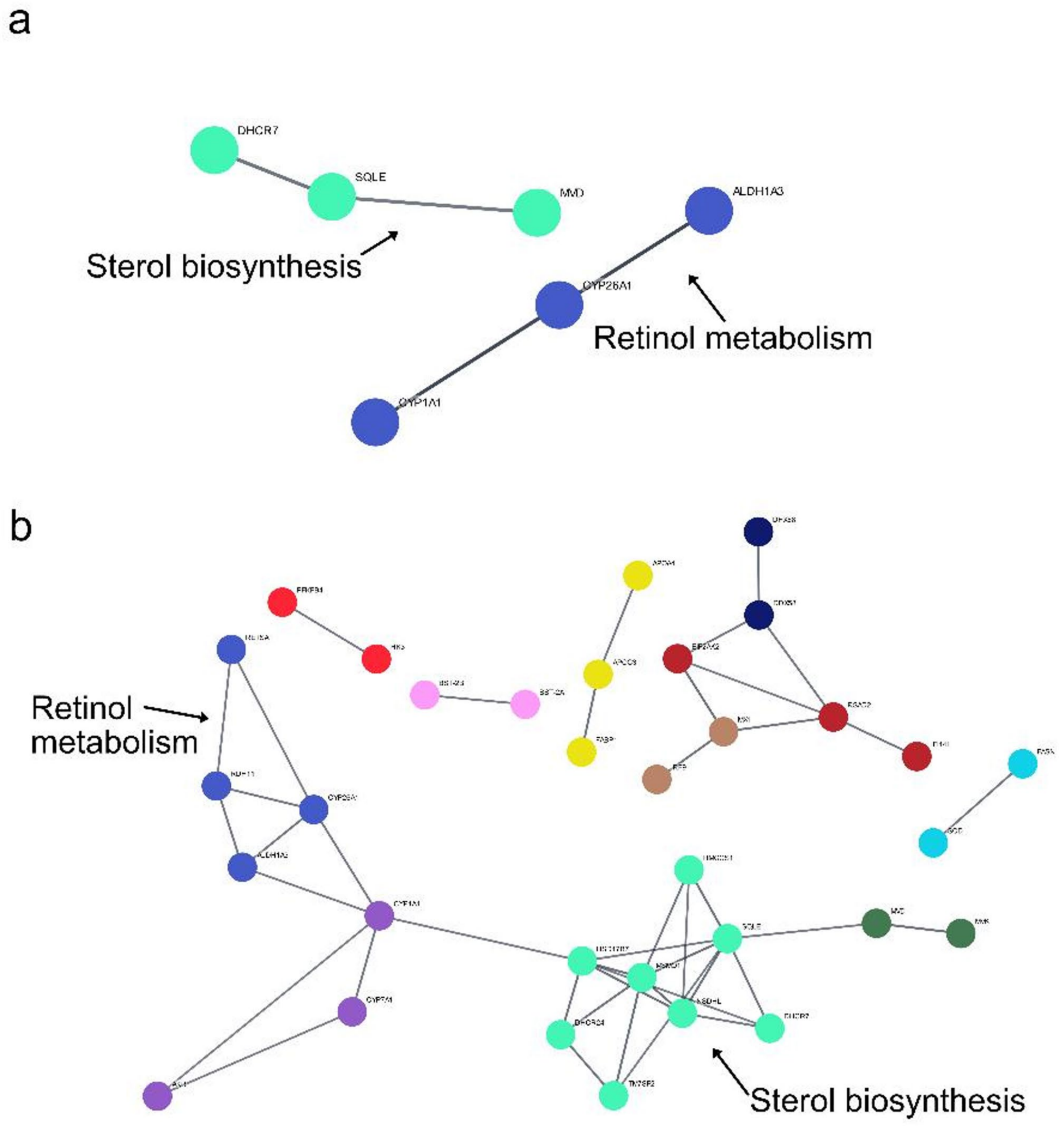
Protein–protein interaction network of DEGs associated with variation in liver copper indicating the two jointly identified clusters for datasets 1 (**a**) and 2 (**b**). Genes within a cluster are indicated by a similar node colour. Edges represent high confidence (0.9) interactions.

After mapping and quality control, 30,186 expressed genes were available for differentially expressed genes (DEGs) analysis. In this study, analysis of dataset 1 revealed that 120 genes were differentially expressed for HLC vs LLC groups, whereas 421 genes were differentially expressed between the groups in dataset 2. However, differences were significant only for 64 and 252 genes at FDR ≤ 0.05 (adjusted P-value) and |log2foldchange|≥ 1 (Supplementary Tables S4a and S4b) for datasets 1 and 2, respectively. Of these DEGs, 31 and 33 DEGs (Fig. 2a, Supplementary Table S4a) were significantly upregulated and downregulated in HLC samples for datasets 1, respectively. On the other hand, 103 DEGs and 149 DEGs (Fig. 2b, Supplementary Table S4b) were upregulated and downregulated in the HLC group for datasets 2, respectively. For clarity, all significantly upregulated genes in the HLC group were downregulated in the LLC group, and vice versa. For both datasets, the volcano plots show the significantly upregulated or downregulated genes associated with the HLC group (Fig. 2a and b, Supplementary Tables S4a and S4b), whereas the heatmap plots reveal the degree of expression for significantly up- or downregulated DEGs between the HLC and LLC groups in this study (Fig. 3a and b). A total of 53 significant DEGs were jointly identified for both datasets. The results of functional enrichment analysis for dataset 1 show that 11 GO and 3 KEGG pathway terms were significantly enriched at *P*-value ≤ 0.05. Additionally, 2 GO and 1 KEGG pathway terms were significantly enriched at FDR ≤ 0.05 (Fig. 4a). Regarding dataset 2, our results show that 41 GO and 20 KEGG pathway terms were significantly enriched at P-value ≤ 0.05 with 14 (8 GO and 6 KEGG pathway) terms significantly enriched after P-value adjustment at FDR ≤ 0.05 (Fig. 4b). Furthermore, the Protein–protein interaction (PPI) network analysis revealed 2 and 9 clusters of interacting DEGs for datasets 1 and 2, respectively (Fig. 5a and b, Supplementary Table S5). Two PPI clusters were jointly identified using both datasets.

### Validation of RNAseq data by RT-qPCR

**Fig. 6:**
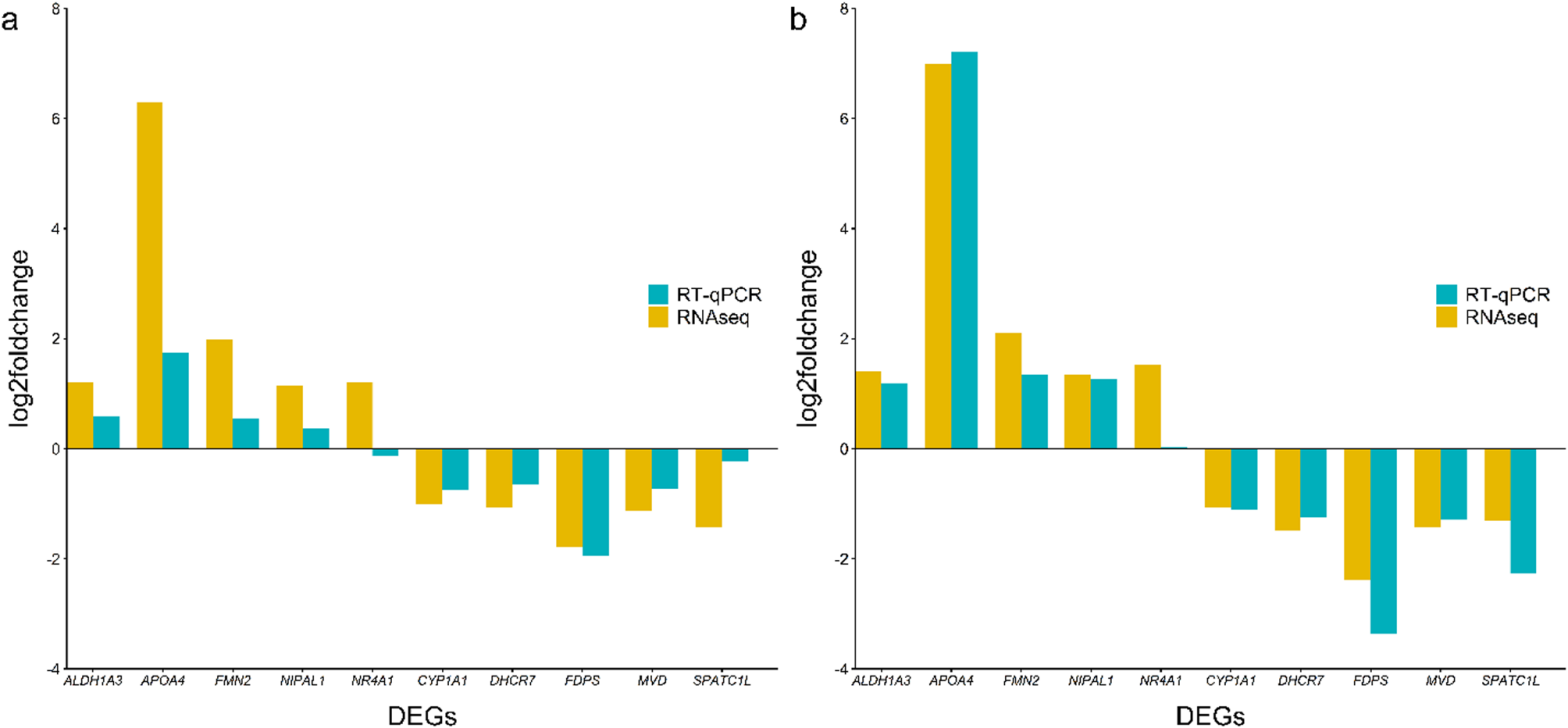
Comparison of reverse transcription-quantitative polymerase chain reaction (RT-qPCR) and RNAseq measured expression of selected significantly (FDR ≤ 0.05; |log2foldchange|≥ 1) up- or down-regulated differentially expressed genes (DEGs) in the high liver copper (HLC) group for datasets 1 (**a**) and 2 (**b**).

A selection of differentially expressed genes observed in both LC groups and datasets using RNAseq data was validated by reverse transcription-quantitative polymerase chain reaction (RT-qPCR). For dataset 1, the direction of the gene expression was observed to be similar to that detected with RNAseq data for all genes (*APOA4, FMN2, NIPAL1, ALDH1A3, CYP1A1, SPATC1L, FDPS, MVD, DHC*R7) with the exception of *NR4A1* (Fig. 6a). This exception was not observed for dataset 2 with the direction of the gene expression found to be like the RNAseq data expression (Fig. 6b). Furthermore, the magnitude of the gene expression for both methods was observed to be similar in some genes for both datasets. The similarity in magnitude of the gene expression improved in dataset 2 when compared to dataset 1 (Fig. 6a and b).

## Discussion

This study evaluated differences in gene expression of hepatic tissue with high and low liver Cu concentration in Polish Merino sheep. As expected, variation in hepatic Cu levels was evident in the sampled sheep reared under similar conditions as seen in Table 1. An earlier study in Merino sheep kept under similar rearing conditions by Judson et al.^8^ found values of 6.7 and ± 2.6 as mean and standard deviation of liver Cu, respectively. Findings by Judson et al.^8^ equate to a 37.3% coefficient of variation for the liver Cu values and supports evidence of within-breed variation in liver Cu concentration in sheep. Additionally, our result supports recent findings by Adeniyi et al.^7^ on within-breed variations in liver Cu accretion in Merinoland sheep when kept under similar feeding and environmental conditions. On average, 93.84 and 93.99% of the clean reads were uniquely mapped to the ovine reference genome (NCBI: *Ovis aries*, ARS-UI_Ramb_v2.0, annotation release 105) for the LLC and HLC groups, respectively. This indicates that the transcriptome sequencing quality is good. Some samples selected for transcriptome analysis did not clearly separate in a PCA plot (Fig. 1a) according to LC groups. Therefore, a subset of dataset 1 (dataset 2) was reanalyzed with better clustering in the PCA plot (Fig. 1b). A comparison of the DEG analyses with the two different data sets show that the number of significant DEGs was lower for dataset 1 (n = 64) when compared to dataset 2 (n = 252) suggesting an increase in significant DEGs discovery. This may be due to higher similarity in expression patterns of samples in each LC groups for dataset 2 as evidenced by the better sample clustering seen in the second heatmap plot (Fig. 3b). The selected significant DEGs observed with RNAseq data were successfully validated using RT-qPCR.

In this study, the expression of 33 and 149 genes was significantly downregulated in the HLC group for datasets 1 and 2, respectively. Of these, 5 downregulated genes jointly identified for both datasets are directly involved in the cholesterol biosynthesis pathway including mevalonate diphosphate decarboxylase (*MVD*), squalene epoxidase (*SQLE*), 7-dehydrocholesterol reductase (*DHCR7*), farnesyl diphosphate synthase (*FDPS*) and lanosterol 14-alpha demethylase (*LOC101113583*)^21,22^. The MVD, FDPS, SQLE, lanosterol 14-alpha demethylase and DHCR7 enzymes catalyze the production of isopentenyl-5-pyrophospate, farnesyl-pyrophosphate, 2,3-oxidosqualene, 4,4-dimethyl-5alpha-cholesta-8,14,24-trien-3beta-ol and cholesterol, respectively, within the cholesterol biosynthetic pathway. In addition to this, many more cholesterol-related genes including mevalonate kinase (*MVK*), 3-hydroxy-3-methylglutaryl-CoA synthase 1 (*HMGCS1*) and NAD(P) dependent steroid dehydrogenase-like (*NSDHL*) encoding enzymes that produce mevalonate-5-phosphate, 3-hydroxy-3-methylglutaryl-CoA and cholesta-8(9)-en-3beta-ol, respectively, were significantly downregulated for dataset 2. An association between Cu concentration and cholesterol biosynthesis has been suggested in earlier studies^23–25^. In these studies, it was observed that cholesterol biosynthesis was downregulated in ATP7B-deficient mice resulting from increased copper levels. Likewise, reviews by other authors indicate that a possible relationship exist between Cu homeostasis and lipid metabolism^26–28^. Similarly to the cholesterol synthesis-related genes, the ergosterol biosynthetic protein 28 homolog (*LOC101110679*) gene was downregulated in the HLC group. A recent study by Capell-Hattam et al.^29^ shows the involvement of ERG28 protein in cholesterol synthesis via activation of SREP2. Moreover, we also identified the FUN14 domain-containing protein 2 (*LOC114118711*) gene, known to aid triglyceride homeostasis via *SREBP1c* upregulation^30,31^, as a downregulated gene in the HLC group. In line with the review of earlier studies by Blades et al.^27^, our results confirm that cholesterol biosynthesis is upregulated in hepatocytes with low Cu concentration and downregulated in hepatic cells with high Cu concentration, respectively.

Furthermore, this study identified the insulin induced gene 1 (*INSIG1*) as an upregulated gene in the LLC group. The INSIG1 protein has been identified as an important protein involved in the regulation of cholesterol synthesis via sterol regulatory element-binding proteins (SREBPs) and 3-hydroxy-3-methylglutaryl coenzyme A reductase (HMG-CoA reductase)^32^. The SREBPs are membrane bound transcription factors that modulate the expression of genes involved in lipid synthesis, while HMG-CoA reductase is a rate-limiting enzyme involved in the cholesterol synthesis pathway^22,33^. The INSIG1 protein forms a complex with SREBP cleavage-activating protein (SCAP) and SREBPs in the endoplasmic reticulum (ER), leading to the inhibition of cholesterol synthesis occasioned by preventing SREBPs transport to the Golgi apparatus^32,34^. Likewise, INSIG1 protein binds directly to HMG-CoA reductase resulting in the accelerated degradation of the enzyme followed by suppression of cholesterol synthesis^32^. Though INSIG1 functions in the reduction of cholesterol levels, it is possible that the significantly higher expression of *INSIG1* gene observed in the LLC group indicates a feedback response to high cholesterol levels in the hepatocytes. In this study, we noted that these cholesterol-related genes were significantly downregulated in the HLC group for both datasets. This suggests that genes associated with cholesterol synthesis are downregulated in liver cells with high liver Cu concentration and upregulated in hepatocytes with low liver content. However, these results do not explain the cause for within-breed variation in hepatic Cu levels in the liver samples because changes in cholesterol biosynthesis are considered an outcome of changes in Cu concentration levels as reviewed by Blades et al.^27^ and Engle^28^.

Four genes involved in retinoic acid metabolism including aldo-keto reductase family 1 member B10 (*LOC101107697 or AKR1B10*), cytochrome P450 26A1 (*LOC101103439 or CYP26A1*), aldehyde dehydrogenase 1 family member A3 (*ALDH1A3*) and nuclear receptor 4A1 (*NR4A1*) genes^35,36^ were significantly differently expressed. Of these, *AKR1B10* and *CYP26A1* were downregulated, while *ALDH1A3* and *NR4A1* were upregulated in the HLC group. Retinoic acid has been associated with intrinsic apoptotic pathways that occur after mitochondrial permeation occasioned by the modulation of proapoptotic (e.g. Bax) and anti-apoptotic (e.g. Bcl-2) proteins that mediate mitochondrial-based apoptosis^37^. Additionally, retinoic acid has also been indicated in the regulation of the expression of caspases that mediate inflammatory responses and apoptosis^37,38^.

AKR1B10 catalyzes the reversible step in retinoid metabolism by converting retinal (substrate for retinoic acid production) to retinol, whereas CYP26A1 regulates retinoic acid levels by conversion to its hydroxy- and/or oxo-metabolites^36,39^. These findings suggest that AKR1B10 and CYP26A1 assist with the reduction of retinoic acid concentration with the attendant effect on retinoic acid signaling. Conversely, the aldehyde dehydrogenase 1 family member A3 (*ALDH1A3*) and nuclear receptor 4A1 (*NR4A1*) genes associated with retinoic acid production and signaling were upregulated in the HLC group. ALDH1A3 is an enzyme that catalyzes the conversion of retinal to retinoic acid leading to increased retinoic acid production^40^, whereas NR4A1 is a transcription factor for the regulation of gene expression associated with retinoic acid signaling^39^. Notably, studies have shown that NR4A1 induces mitochondrial related apoptosis by targeting Bcl-2 located on the mitochondria^41,42^, and interacts with another gene that was upregulated in the HLC group in this study known as apolipoprotein A4 (*APOA4*). APOA4 downregulates gluconeogenesis in mice and human hepatocytes^43,44^. Thus, genes associated with an increase in retinoic acid synthesis and signaling were upregulated in the HLC group, while genes associated with decreased retinoic acid synthesis were downregulated.

Similar to our observation regarding cholesterol biosynthesis and retinoic acid activity, a report by Muchenditsi et al.^45^ on gene expression in *ATP7B* deficient mice showing the Wilson disease phenotype with increased Cu accumulation found that cholesterol biosynthesis and liver X receptor/retinoid X receptor (LXR/RXR) activation were downregulated and upregulated, respectively. Furthermore, the spermatogenesis and centriole associated 1 like (*SPATC1L*) gene located 148.7 Kilobases (Kb) from a SNP (rs427314005) that was identified in our previous genome-wide association study on genes influencing liver Cu concentration in Merinoland sheep was downregulated in the HLC group^7^. The SPATC1L protein reportedly associates with the regulatory subunit of protein kinase A, whereas its involvement in Cu homeostasis is unknown. A look at the recent sheep genome annotation (Ovis aries ARS-UI_Ramb_v2.0 genome assembly in NCBI) reveals that a gene known as lanosterol synthase (*LSS*) is located approximately 35 Kb from the *SPATC1L* gene. This implies that both genes are linked within the genome, though no interaction has been established between their proteins. The *LSS* gene has been implicated in the catalysis of a reaction that produces a substrate within the cholesterol biosynthesis pathway named lanosterol. This suggests that the *SPATC1L* and *LSS* genes may be associated with variation in liver copper accumulation in Polish Merino sheep. Interestingly, the *LSS* gene was differentially expressed at FDR ≤ 0.05 in this study but not significant due to a |log2foldchange| less than 1 (|log2foldchange| for *LSS* = 0.999). Further studies need to be conducted to ascertain the effect or not of these genes on hepatic Cu levels.

In this study, some significantly enriched GO terms including cholesterol biosynthetic process (GO:0006695), endoplasmic reticulum membrane (GO:0005789) and cholesterol metabolic process (GO:0008203) were jointly identified for both datasets with involved genes including *INSIG1*, *APOA4*, *MVD* and *DHCR*. Besides, some KEGG pathway terms including retinol metabolism (oas00830), metabolic pathways(oas01100) and steroid biosynthesis (oas00100) were enriched for both datasets in this study. Genes such as *LOC101107697, ALDH1A3* and *LOC101103439* were involved in the enrichment of these KEGG pathway terms. Other significantly enriched KEGG pathway terms identified for dataset 2 in this study include PPAR signaling pathway (oas03320) and cholesterol metabolism (oas04979). In agreement with the gene enrichment analysis, sterol biosynthesis and retinol metabolism were the primary functions of the two PPI network clusters jointly identified for both datasets. Genes such as *DHCR7* and *SQLE* were included in the cluster for sterol biosynthesis, while *ALDH1A3* and *CYP26A1* were part of the cluster for retinol metabolism. Our findings suggest that cholesterol and retinol metabolism processes are associated with variations in hepatic Cu levels in Polish Merino sheep. This study has some limitations such as the relative low number of samples used in transcriptome analysis as well as the slight variation in age at slaughter (included as a fixed effect in the DEG analysis) among the samples. Additionally, an inclusion of male lambs in a new study may aid our understanding on sex influence on gene expression associated with within-breed differences in hepatic Cu concentration.

## Conclusion

A total of 53 significantly up- or downregulated genes were jointly observed for both datasets. Of these, we identified that some downregulated genes in the HLC group including *MVD*, *SQLE*, *DHCR7*, *FDPS*, *LOC101113583* and *ERG28* are associated with cholesterol synthesis. Likewise, this study revealed 4 genes including *AKR1B10, CYP26A1*, *ALDH1A3* and *NR4A1* that are implicated in retinoic acid synthesis. Moreover, GO and KEGG pathway enrichment highlighted processes involving cholesterol biosynthesis, endoplasmic reticulum membrane, steroid biosynthesis and retinol metabolism. A gene (*SPATC1L*) located near a SNP identified in our previous study was significantly downregulated in the HLC group. Our findings deliver possible candidate genes and processes that may influence or serve as an indicator for difference in within-breed liver Cu concentration in sheep, and may aid in breeding improvements for reduction in the occurrence of Cu intoxication or deficiency in sheep farming.

## Materials and methods

All sampled lambs of the Polish Merino breed were raised for lamb meat production on a commercial farm in Poland. Lambs were slaughtered in a commercial abattoir and carcasses were prepared for sale. Biological samples were collected post-mortem from these carcasses. As no animal was slaughtered for scientific purposes, the responsible Ethics Committee did not classify this study as animal experiment.

### Sample collection

Lambs were raised indoors under similar environmental conditions. All sheep had free access to drinking water and were fed *ad libitum* on the same diet of hay, pellet concentrate, silage and whole oats grains. Lambs were fasted 24 h before slaughter. Samples of liver tissue from the *lobus caudatus* region were collected from 38 lambs immediately after slaughter into FixRNA (Eurx, Gdańsk, Poland) according to manufacturer’s instructions, stored for 24 h in this buffer at 4 °C, and subsequently frozen in cryotubes at − 80 °C for long-term storage. In addition, samples were collected from the *lobus caudatus* region of the liver, and frozen at − 20 °C for the determination of liver Cu content. Sex and ages at slaughter were recorded for all lambs.

### Determination of Cu content in liver and feed samples, grouping of samples

All 38 liver samples were freeze-dried, crushed and stored in tubes. Analysis of liver Cu concentration was performed with an inductively coupled plasma-optical emission spectrometer (ICP-OES; Agilent 720ES, Darmstadt, Germany) at a wavelength of 327.4 nm after microwave digestion as described by Adeniyi et al.^7^. Furthermore, mineral concentrations of feed samples were determined by a service laboratory (Intertek Food Services GmbH, Linden, Germany) (Supplementary Table S1). Liver copper concentration values were reported in mg/kg DM. Of the 38 samples analyzed for liver Cu concentration, a subset of 12 samples (dataset 1) from ewes with the lowest (n = 6) and highest (n = 6) Cu concentration were selected into two liver Cu (LC) groups (LLC = low liver Cu; HLC = high liver Cu) for RNA sequencing and analysis.

### RNA extraction

Total RNA was extracted from the selected liver samples (dataset 1; n = 12) using a Universal RNA Purification Kit (Eurx, Gdańsk, Poland) according to the manufacturer’s protocol for animal tissues with the use of RNA Extracol reagent (Eurx, Gdańsk, Poland). RNA quantity and purity were assessed on a Nanodrop 2000 spectrophotometer (Thermo Scientific, Waltham, MA, USA). RNA integrity was evaluated on an Agilent Bioanalyzer 2100 system (Agilent Technologies, Santa Clara, CA, USA) using an RNA Nano 6000 Assay Kit RNA (Agilent Technologies, Santa Clara, CA, USA). RNA degradation and contamination were monitored on 1% agarose gel. All the samples passed the quality control requirements (RNA integrity number (RIN) ≥ 7.5) and were processed for downstream applications.

### RNA sequencing and cDNA mapping

Firstly, ribosomal RNA was removed from total RNA using TruSeq Stranded Total RNA Library Prep (Illumina, San Diego, CA, USA). RNA-seq libraries were prepared using Novogene NGS Stranded RNA Library Prep Set (PT044). All cDNA libraries were sequenced using a paired-end strategy with a reading length of 150 bps on an Illumina NovaSeq 6000 sequencing platform (Illumina, San Diego, CA, USA) at a depth of 50 million reads per sample by Novogene (Beijing, China). These raw data were processed using fastp v0.20.1 (https://github.com/OpenGene/fastp)^46^ to remove adapter sequences and trim low-quality reads to obtain clean data for downstream analysis. The paired-end reads that passed the quality control were mapped to the sheep reference genome (NCBI: *Ovis aries*, ARS-UI_Ramb_v2.0, annotation release 105) using STAR v2.7.9a aligner (https://github.com/alexdobin/STAR)^47^.

### Principal component analysis

Using the count matrix, a principal component analysis (PCA) was performed with dataset 1 using prcomp package in R v3.3.3 software (https://www.R-project.org/)^48^ after count matrix data transformation with the *rlog* function in R “DESeq2” package^49^. Subsequently, a PCA plot comparing the first principal component (PC 1) against PC 2 was drawn with the ggplot package in R. Due to incomplete clustering of the samples, a subset of dataset 1 (dataset 2) consisting of 6 individuals (LLC: n = 3; HLC: n = 3) with the best possible clustering of samples into LC groups according to PC 1 of the first PCA plot was selected. As described for dataset 1, a second PC analysis was performed for dataset 2 with the construction of a PCA plot showing the first two PCs.

### Statistical analysis

A t-test and Mann-Whitney U test to compare means of LC groups for liver Cu concentration and age were performed for dataset 1 using the *t.test* and *wilcox.test* functions in R v3.3.3 “stats” package, respectively^48^. Prior to this, data on liver Cu concentration and age were tested for fulfillment of parametric test assumptions using the *shapiro.test* and *var.test* functions in the above-mentioned package in R with the LC groups data for liver Cu concentration fulfilling all parametric test assumptions whereas data for age did not. No statistical analysis was carried out for dataset 2 due to low sample size. Descriptive statistics of all samples and LC groups of datasets 1 and 2 were calculated using the dplyr package in R^50^.

### Analysis of differentially expressed genes (DEGs analysis)

Using dataset 1, a comparison of the reads per gene for both LC groups was analysed for determination of differentially expressed genes (DEGs) using DESeq2 package in R v3.3.3^49^. The DEGs analysis of HLC vs LLC was performed with a generalized linear model that included age as fixed effect with two categories (less or more than 300 days, respectively). This was implemented in the DESeq2 package with the function *DESeqDataSetFromMatrix* (design: ~ age + LC_Groups). The DEGs were identified as significant at FDR ≤ 0.05 (adjusted *P*-value)^51^ and |log2foldchange|≥ 1^20^. This log2foldchange threshold was selected in order to account for small biological but meaningful changes in expression that may occur between both LC groups, considering that hepatic Cu levels of most (4 samples of 6) of the samples for each groups were within normal physiological liver Cu levels for sheep (100–400 mg Cu/Kg DM)^52^, and may result in a slight increase in the number of identified DEGs. Result of the DEGs analysis was used in the construction of a volcano plot to show significantly up- or down regulated DEGs using ggplot2 package in R^53^. Furthermore, a heatmap plot showing differences in the expression profile of significantly up- or down regulated DEGs for LLC and HLC samples was drawn using heatmap3 package in R^54^. Prior to the construction of the heatmap plot, count data was normalized using the *vst* function in R “DESeq2” package. Using similar parameters, DEGs analysis and heatmap plotting was repeated for dataset 2 with the results of DEGs analysis plotted in a volcano plot.

### DEGs identification, enrichment and PPI network analyses

Differentially expressed genes identified as significant in the analyses of datasets 1 and 2 were separately included in functional enrichment analysis using the Database for Annotation, Visualization and Integrated Discovery (DAVID v2023q4) software (https://david.ncifcrf.gov, accessed on 12 September 2024)^55–57^. The gene list was analyzed by selecting the ovine gene annotations. The gene ontology (GO) and pathways terms including biological process (BP), cellular component (CC), molecular function (MF) and Kyoto encyclopedia of genes and genomes (KEGG)^58^, were investigated in this study. Enriched GO and KEGG pathway terms were considered significant at P-value ≤ 0.05 and FDR ≤ 0.05^51^ before and after correction for multiple testing, respectively. Using the search tool for the retrieval of interacting genes (STRING v12.0: https://string-db.org/) with a high confidence interaction score of 0.9^59^, protein-protein interaction (PPI) network analysis was performed for identified DEGs for both datasets, separately. The result from the PPI analysis was viewed using Cytoscape v3.10.2 (https://cytoscape.org/)^60^.

### Reverse transcription-quantitative PCR analysis

From the jointly identified DEGs and genes involved in enriched pathways considered significant in both datasets, a total of 10 DEGs (5 upregulated and 5 downregulated) were selected for RT-qPCR. From extracted RNA, first-strand cDNAs were synthesized using smART First Strand cDNA Synthesis kit (Eurx, Gdańsk, Poland) according to the manufacturer’s instructions. SYBER Green chemistry was used to measure the gene expression levels on a Bio-Rad CFX Opus 96 Real-Time PCR System. The primers of DEGs selected for validation were designed using Primer-BLAST software (https://www.ncbi.nlm.nih.gov/tools/primer-blast/index.cgi, accessed on 10th June, 2024)^61^. The glyceraldehyde-3-phosphate dehydrogenase gene (*GAPDH*) and actin beta (*ACTB*) genes were used as endogenous controls with primer information sourced from Schulze et al.^62^ and French et al.^63^, respectively. The qPCRs were performed in triplicate in a final volume of 20 µl containing 50 ng cDNA, 10 µl SG qPCR Master Mix (Eurx, Gdańsk, Poland), 5 pmol of each primer (forward and reverse) (Supplementary Table S2), 0.2 U uracil-N-glycosylase (UNG), and nuclease-free water. The qPCR program was started with UNG pre-treatment at 50 °C for 2 min., followed by initial denaturation at 95 °C for 10 min., 40 cycles of 94 °C for 15 s, 60 °C for 30 s, and 72 °C for 30 s, and ended with a final stage of melting curve analysis, which was carried out using following conditions: 94 °C for 5 s, 70 °C for 5 s and then a gradual increase in temperature to 95 °C at a ramp rate of 0.5 °C/5 s to ensure that single product was amplified in each reaction. All qPCR reactions showed a single peak on the dissociation curve confirming the specific amplification of PCR products. The average cycle threshold (Ct) values of the genes were normalized to the geometric means of the controls. The delta delta Ct method was used to analyze the relative expression levels of the studied genes. Then, the log2foldchange was calculated from the relative expression.

## Electronic supplementary material

Below is the link to the electronic supplementary material.

Supplementary Material 1

## Data Availability

All sequence data generated during this study has been deposited to the ArrayExpress collection in BioStudies with the accession number E-MTAB-14752 (https://www.ebi.ac.uk/biostudies/arrayexpress/studies/E-MTAB-14752?key=5ea77d94-615d-47e7-822f-5d20fcc34ea5).
